# Economic burden of comorbid chronic conditions among survivors of stroke in China: 10-year longitudinal study

**DOI:** 10.1186/s12913-021-07010-1

**Published:** 2021-09-17

**Authors:** Ji Zhang, Suhang Song, Yang Zhao, Gaoting Ma, Yinzi Jin, Zhi-Jie Zheng

**Affiliations:** 1 Department of Global Health, School of Public Health, Peking University, 38 Xue Yuan Road, Haidian District, Beijing, 100191 China; 2 Institute for Global Health and Development, Peking University, Beijing, China; 3 Taub Institute for Research in Alzheimer’s Disease and the Aging Brain, Columbia University, New York, NY USA; 4 The Nossal Institute for Global Health, Melbourne School of Population and Global Health, The University of Melbourne, Melbourne, VIC 3010 Australia; 5 WHO Collaborating Centre on Implementation Research for Prevention and Control of Noncommunicable Diseases, Melbourne, VIC 3010 Australia; 6 Department of Interventional Neuroradiology, Beijing Tiantan Hospital, Affiliated to Capital Medical University, Beijing, China

**Keywords:** Stroke, Comorbidities, Economic burden, Hypertension, Diabetes mellitus, Cognitive impairment

## Abstract

**Background:**

The coexistence of chronic diseases among people with stroke is common. However, little is known about the extent of incremental healthcare expenditures associated with having physically and psychologically chronic conditions among stroke survivors.

**Methods:**

We used the nationally representative data from the China Health and Nutrition Survey, including 36,076 participants enrolled as our analytic cohort of ten years of follow-up visits (2006, 2009, 2011, 2015). Chronic conditions include hypertension, diabetes, obesity, and impaired cognitive function. Two-part models were used to estimate the effect of comorbid chronic conditions on total annual healthcare expenditure, out-of-pocket (OOP) healthcare expenditure, and incidence of catastrophic healthcare expenditure (CHE).

**Results:**

Among survivors of stroke during 2006 to 2015, the prevalence rates of hypertension, diabetes, obesity and impaired cognitive function were 75.5, 9.8, 12.7 and 65.1%, significantly higher than those among adults without stroke history (27.9, 2.7, 10.0 and 41.2%). Having hypertension ($794.5, *p* = 0.004), diabetes ($3978.5, *p* < 0.001) were associated with the largest incremental total healthcare expenditures. Stroke survivors with diagnosed hypertension and diabetes had additional 5.7 (p < 0.001) and 10.4 (p < 0.001) percentage point of CHE rate, respectively. Total healthcare expenditures were $2413.0 (*P* < 0.001) and $5151.7 (P < 0.001) higher among patients with 2, and ≥ 3 chronic conditions, respectively, than those individuals with no chronic conditions.

**Conclusions:**

Excess expenditures associated with chronic diseases were substantial among stroke survivors. These results highlight the needs for both prevention and better management of multimorbidity among stroke survivors, which in turn may lower the financial burden of treating these concurrent comorbidities.

## Introduction

Stroke is the leading cause of death in China, which accounted for about one-third of worldwide stroke mortality [1]. Stroke requires emergent and expensive care, and over 70% of stroke survivors in China experienced catastrophic out-of-pocket (OOP) expenditure due to loss of income and cost of health care [2]. The economic burden of stroke is increasing on aging populations in China. A notable trend among the stroke survivors is the rising coexistence with chronic conditions, including cardiovascular and psychological conditions [3]. Such coexistence imposes a considerable financial burden on individuals and families, as a result of increased healthcare use and costs, work productivity loss, and reduced income.

Although the financial burden of stroke among the general population has been studied, no study, to our knowledge, has analyzed the concurrent chronic conditions in stroke survivors and their related economic burden, in low- and middle-income countries [2, 4–7]. Understanding the prevalence and burden of comorbidities in stroke survivors is an important consideration for national efforts to improve care and cost containment in this population. This information can also help optimize health and quality of life among the growing population of people with multiple (two or more) chronic conditions.

To fill this gap, this study aimed to examine the prevalence of comorbid chronic conditions among stroke survivors and those without a history of stroke, and to estimate the effect of comorbidities on medical care costs and incidence of catastrophic health expenditures among survivors of stroke in China.

## Methods

### Study population

Participants were enrolled in the China Health and Nutrition Survey (CHNS), an ongoing cohort longitudinal survey of ten waves (1989–2015) of Chinese adults aged ≥18 years. This study includes assessments of economic, sociological, and health circumstances of community residents in China. The survey employs a multistage random cluster sampling process to draw 4400 households from 361 communities in 15 provinces/municipal cities. This study used data from ten years of four follow-up visits (2006, 2009, 2011, 2015), which covered the period before and after the China health system reform of 2009. Of 49,861 observations, 13,785 observations were excluded due to lack of data, and 36,076 observations were enrolled as our analytic cohort, of which 100% were followed during the ten years of study period. The final protocol of CHNS was approved by the Ethical Review Committee of Chinese Center for Disease Control and Preventive (No. 201524). It was not appropriate or possible to involve survey subjects in the design, or conduct, or reporting, or dissemination plans of our research.

### Measures

#### Definitions of stroke and chronic conditions

The diagnosis of stroke was based on self-report, defined as ever having been or currently diagnosed by a hospital as stroke. Chronic conditions in our study include hypertension, diabetes, obesity, and impaired cognitive function.

Hypertension was defined by a combination of self-reported data and measured blood pressure. Respondents with systolic blood pressure ≥ 140 mmHg and/or diastolic blood pressure ≥ 90 mmHg and/or currently taking anti-hypertensive medication were defined as hypertensive. Diabetes was defined as self-reported history of diabetes or currently taking anti-diabetic drugs. Obesity was defined as the BMI ≥28 by measuring height without shoes in meter and weight in kilogram to calculate the BMI (kilograms per square meter).

Cognitive function was tested in the year of 2006 and 2015. This study used a global cognitive function score based on two domains of cognitive functions: word recall and numerical ability. For word recall, each respondent was asked to repeat as many of the 10 Chinese nouns just read to them as possible (immediate word recall) and then to recall the same 10-word list 5 min later (delayed recall). Answers to these questions were aggregated into a single word recall score ranging from 0 to 10. Numerical ability was measured by counting backwards from 20 to 1 for twice (1 point for one time is correct) and serial 7 subtractions from 100 (up to 5 times). Answers to these questions were then aggregated into a single numerical ability score ranging from 0 to 7. Respondents with global score ≤ 10 points would be defined with impaired cognitive function [8, 9].

#### Healthcare expenditure and catastrophic healthcare expenditure

Respondents were asked about their healthcare expenditures and health insurance reimbursement rate, in the past 4 weeks. Total healthcare expenditures were calculated on the basis of year, including all direct medical costs such as physical examinations, medical treatments, pharmaceuticals, and direct nonmedical costs such as transportation fee. OOP healthcare expenditures were calculated accordingly, and those of ≥30% of the total household income were defined as catastrophic healthcare expenditure (CHE) [10].

#### Other individual and household characteristics

Based on prior studies, several covariates were included in the present study [11–13]. They include demographic and socioeconomic status covariates such as age (< 55 or ≥ 55 years), gender (male or female), marital status (single, married, divorced/widowed/ separated, or refuse to answer), educational attainment (illiterate/primary school, junior school, high school or above), household income per capita (lower, middle or upper tertile), employment (employed or not), health insurance (Urban Employee Basic Medical Insurance, Urban Resident Basic Medical Insurance, New Rural Cooperative Medical Insurance, commercial and other insurance, or none of above). We also included the area of residence (city, suburban, town, or village) and the location of residence (northern or southern) in analyses of environmental variables at the community level.

### Statistical analysis

A descriptive analysis presented the prevalence of multiple chronic conditions, total annual healthcare expenditures, OOP healthcare expenditures, and incidences of CHE among the full sample as a whole and then by subsamples of stroke survivors and those without a history of stroke. Two pairs of two-part models were used to estimate the effect of multiple chronic conditions on total annual healthcare expenditures, OOP healthcare expenditures and CHE incidences, given the large number of respondents without any use of healthcare services. The first pair of models focused on evaluating incremental healthcare expenditures for each of the chronic conditions by separate models, and the second pair of models focused on number of chronic conditions as independent predictors. Healthcare expenditures were estimated using generalized linear models with a gamma distribution and a log link. Incidence of CHE was estimated using generalized linear models with a negative binomial distribution and a log link. We used analysis of variance for univariate analysis of means for the comparison of means, the Wilcoxon rank test for the comparison of medians, and chi-square test for the comparison of frequencies. We used SAS (version 9.4, SAS Institute Inc., Cary, NC, USA) in all statistical analyses.

## Results

### Prevalence of comorbid chronic conditions

Compared with individuals without a history of stroke, survivors of stroke had higher proportion of individuals aged≥55 years (88.6% vs 39.8%), higher percentage of lower household income per capita (42.4% vs 34.9%), higher percentage of having primary/illiterate education (61.1% vs 40.8%), and lower rate of being employed (17.7% vs 57.4%). Among survivors of stroke during 2006 and 2015, the prevalence of hypertension, diabetes, obesity and impaired cognitive function were 75.5%, 9,8, 12.7 and 65.1%, significantly higher than those among adults without stroke history (27.9, 2.7, 10.0 and 41.2%) in China (Table 1). Stroke survivors had higher prevalence of hypertension, diabetes, and impaired cognitive function than those without stroke history at each wave. Among stroke survivors, 72.2 and 4.2% had hypertension and diabetes in 2006, while in 2015, the prevalence rates increased to 79.7 and 16.3%, respectively (Table 2).

**Table 1:** Demographic characteristics of survivors of stroke: CHNS 2006–2015

Characteristic	Total		Stroke survivors		Non-stroke		*P* value
	N	%	N	%	N	%	
Total	36,076	100.0	458	100.0	35,618	100.0	
Male	17,285	47.9	297	64.8	16,988	47.7	<.0001
Age group							
< 55 years	21,479	59.5	52	11.4	21,427	60.2	<.0001
≥ 55 years	14,597	40.5	406	88.6	14,191	39.8	
Married							
Single	2353	6.5	3	0.7	2350	6.6	<.0001
Married	30,112	83.5	367	80.1	29,745	83.5	
Divorced/widowed/separated	3474	9.6	86	18.8	3388	9.5	
Refuse to answer	137	0.4	2	0.4	135	0.4	
Education level							
Primary/illiterate	14,810	41.1	280	61.1	14,530	40.8	<.0001
Junior school	11,905	33.0	97	21.2	11,808	33.2	
High school /above	9361	26.0	81	17.7	9280	26.1	
Household income per capita							
Lower tertile	12,621	35.0	194	42.4	12,427	34.9	0.0033
Middle tertile	11,993	33.2	130	28.4	11,863	33.3	
Upper tertile	11,462	31.8	134	29.3	11,328	31.8	
Employed	20,537	56.9	81	17.7	20,456	57.4	<.0001
Medical Insurance							
Non	6332	17.6	69	15.1	6263	17.6	0.0024
Urban Employee Basic Medical Insurance	6427	17.8	112	24.5	6315	17.7	
Urban Resident Basic Medical Insurance	3561	9.9	42	9.2	3519	9.9	
New Rural Cooperative Medical Insurance	18,536	51.4	226	49.3	18,310	51.4	
Commercial and other insurance	1220	3.4	9	2.0	1211	3.4	
Urbanization stratum							
City	5034	14.0	85	18.6	4949	13.9	0.002
Suburban	6744	18.7	67	14.6	6677	18.7	
Town or county capital	5786	16.0	87	19.0	5699	16.0	
Rural village	18,512	51.3	219	47.8	18,293	51.4	
Region							
North	15,295	42.4	234	51.1	15,061	42.3	0.0002
South	20,781	57.6	224	48.9	20,557	57.7	
Wave							
2006	8002	22.2	72	15.7	7930	22.3	0.0071
2009	9650	26.8	128	27.9	9522	26.7	
2011	9220	25.6	135	29.5	9085	25.5	
2015	9204	25.5	123	26.9	9081	25.5	
Comorbid chronic conditions							
Hypertension	10,289	28.5	346	75.5	9943	27.9	<.0001
Diabetes	1018	2.8	45	9.8	973	2.7	<.0001
Obesity	3623	10.0	58	12.7	3565	10.0	0.0603
Impaired cognitive function	2862	41.85	112	65.1	2750	41.2	<.0001

**Table 2:** Prevalence of chronic conditions among survivors of stroke: CHNS, 2006–2015

Chronic conditions	Total		Stroke survivors		Non-stroke		*P* value
	N	%	N	%	N	%	
Hypertension							
2006	1740	21.7	52	72.2	1688	21.3	<.0001
2009	2785	28.9	96	75.0	2689	28.2	<.0001
2011	2574	27.9	100	74.1	2474	27.2	<.0001
2015	3190	34.7	98	79.7	3092	34.0	<.0001
Diabetes							
2006	97	1.2	3	4.2	94	1.2	0.0707
2009	264	2.7	12	9.4	252	2.6	0.0002
2011	298	3.2	10	7.4	288	3.2	0.0172
2015	359	3.9	20	16.3	339	3.7	<.0001
Obesity							
2006	599	7.5	10	13.9	589	7.4	0.0381
2009	863	8.9	14	10.9	849	8.9	0.426
2011	1006	10.9	21	15.6	985	10.8	0.0812
2015	1155	12.5	13	10.6	1142	12.6	0.5046
Impaired cognitive function							
2006	1291	48.8	50	80.6	1241	48.1	<.0001
2015	1571	37.4	62	71.0	1509	56.4	<.0001

### Healthcare expenditures and catastrophic healthcare expenditure

**Fig. 1:**
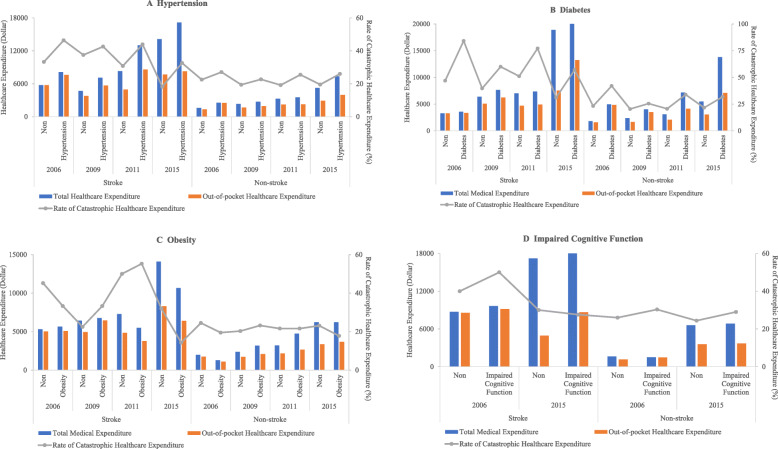
Healthcare expenditures and catastrophic healthcare expenditures among adults with chronic conditions compared with those without chronic conditions for stroke survivors and non-stroke adults: CHNS 2006–2015

In general, the total healthcare expenditures, OOP healthcare expenditures, and CHE incidences among stroke survivors were higher than those among non-stroke respondents. In the group of stroke survivors, those diagnosed with hypertension, diabetes and impaired cognitive function had higher total healthcare expenditures, OOP healthcare expenditures, and CHE incidences at each wave except for rate of CHE in 2015. The healthcare expenditures increased, while the incidences of CHE decreased between 2006 and 2015 for both diagnosed chronic conditions and those without chronic conditions. For instance, among stroke survivors, the total healthcare expenditures and OOP healthcare expenditures of diagnosed diabetes were $3563.1 and $3345.7, and 84.2% had incidence of CHE in 2006; while in 2015, the total healthcare expenditures and OOP healthcare expenditures were $13,814.6 and $7107.5, and 57.0% had CHE incidence (Fig. 1).

Among stroke survivors, having hypertension ($794.5, 95% CI $409.4 to $2117.9, p = 0.004), diabetes ($3978.5, 95% CI $2302.8 to $5654.3, p < 0.001) were associated with the largest incremental total healthcare expenditures. Stroke survivors with diagnosed hypertension and diabetes had additional 5.7 (95% CI 0.035 to 0.079, *p* < 0.001) and 10.4 (95% CI 0.061 to 0.146, p < 0.001) percentage point of CHE rate, respectively. Total healthcare expenditures were $2413.0 (95% CI $1166.8 to $3659.3, P < 0.001) and $5151.7 (95% CI $2586.2 to $7717.3, P < 0.001) higher among patients with 2, and ≥ 3 chronic conditions, respectively, than those individuals with no chronic conditions. Stroke survivors with 2, and ≥ 3 chronic conditions had 3.7 (95% CI 0.013 to 0.061, *p* = 0.003) and 8.5 (95% CI 0.053 to 0.117, *p* < 0.001) increased percentage point of CHE incidence than those with no chronic conditions, respectively (Table 3).

**Table 3:** Additional healthcare expenditures among survivors of stroke with chronic conditions compared with those without chronic conditions: CHNS 2006–2015

Chronic conditions	Total medical expenditure (inflated to 2015) (Dollar)			Out-of-pocket medical expenditure (inflated to 2015) (Dollar)			Rate of catastrophic healthcare expenditure (%)		
	Additional cost	95% CI	*P*	Additional cost	95% CI	*P*	Additional rate	95% CI	*P*
Model 1: Conditions									
Hypertension	794.5	409.4 to 2117.9	0.004	717.5	236.3 to 1198.6	0.003	0.057	0.035 to 0.079	< 0.001
Diabetes	3978.5	2302.8 to 5654.3	< 0.001	2343.8	1400.1 to 3287.5	< 0.001	0.104	0.061 to 0.146	< 0.001
Obesity	466.4	− 844.9 to 1777.8	0.486	148.5	− 590.2 to 887.1	0.694	0.019	0.014 to 0.053	0.253
Impaired Cognitive Function	556.6	− 1839.4 to 2952.6	0.649	463.1	− 769.5 to 1695.6	0.462	0.035	−0.011 to 0.082	0.139
Model 2: Number of conditions									
1	473.3	− 463.1 to 1409.7	0.322	167.8	− 359.6 to 695.3	0.533	0.027	−0.038 to 0.093	0.420
2	2413.0	1166.8 to 3659.3	< 0.001	1416.4	714.5 to 2118.4	< 0.001	0.037	0.013 to 0.061	0.003
≥ 3	5151.7	2586.2 to 7717.3	< 0.001	2776.2	1331.2 to 4221.1	< 0.001	0.085	0.053 to 0.117	< 0.001

Note: Total medical expenditure in model 1: Adjusted R^2^ = 0.1613, AIC = 36,984.7, BIC = 37,427.8

Total medical expenditure in model 2: Adjusted R^2^ = 0.1164, AIC = 13,992.4, BIC = 14,293.3

Out-of-pocket medical expenditure in model 1: Adjusted R^2^ = 0.1012, AIC = 6324.8, BIC = 7239.6

Out-of-pocket medical expenditure in model 2: Adjusted R^2^ = 0.1127, AIC = 5143.8, BIC = 5442.1

Rate of Catastrophic Healthcare Expenditure in model 1: Adjusted R^2^ = 0.1232, AIC = 13,667.1, BIC = 13,711.4

Rate of Catastrophic Healthcare Expenditure in model 2: Adjusted R^2^ = 0.1210, AIC = 18,017.2, BIC = 18,016.7

## Discussion

To our knowledge, this is the first study to estimate the impact of co-occurring chronic conditions on incremental expenditures and CHE among stroke survivors in low- and middle- income countries. This study shows that physically and psychologically chronic conditions are more prevalent among survivors of stroke and are associated with increased healthcare expenditures and catastrophic payments. These findings highlight that increased efforts in post-acute care and rehabilitation are needed for stroke survivors, given their increased risk of developing multiple chronic conditions and ensuing financial burden.

The current findings are consistent with existing studies on estimation of total healthcare expenditures and OOP healthcare expenditures among Chinese population with stroke in 2006 [2]. The relative higher differences found in this study is due to the higher baseline expenditures of the study population, which consisted of people with a long history of stroke who require long-term rehabilitation rather than the stroke patients at discharge in the prior study. In addition, consistent with previous findings, we found associations between stroke and the presence of comorbid hypertension, diabetes, obesity and impaired cognitive function [14–16]. Our study added the value that, among stroke survivors, the prevalence rates of hypertension and diabetes increased more than those among the adults with no history of stroke during the past decade. The results highlight the importance of understanding the biological mechanisms and indicate that stroke comorbid with chronic conditions result in a heavy burden on individuals, households as well as health systems.

A number of previous studies have demonstrated that healthcare expenditures are associated with stroke complications and comorbidities [5, 17, 18]. Hence, it is necessary to understand what drives expenditures in order to further underscore the need for effective prevention and treatment, and well-designed rehabilitation programs to contain stroke-related healthcare costs. Our study adds the evidence on estimation of the additional expenditures due to the four chronic conditions among stroke survivors. We also found that OOP healthcare expenditures for stroke comorbid with chronic conditions increased, although the CHE incidences decreased between 2006 and 2015.

Several potential explanations account for the rising excess healthcare expenditures among stroke survivors comorbid with chronic conditions. First, comorbid stroke may increase the severity of chronic conditions (or vice versa), which would in turn increase direct expenditures because of the excess costs per utilization of treatments [3]. Patients with chronic conditions may incur healthcare expenditures through the treatment of chronic conditions themselves and procedures for related diseases that usually require post-acute care and rehabilitation [19]. A propensity score-matched analysis has shown that stroke survivors with co-occurring hypertension or diabetes had significantly greater inpatient, emergency room, and prescription expenditures compared with those without co-occurrence [16]. Taking diabetes as examples, some previous studies have revealed higher recurrence rates of stroke among patients with diabetes than among those without, and stroke are also more difficult to manage when these conditions coexist with diabetes [20, 21]. The higher healthcare expenditures associated with per inpatient stay and per outpatient visit could be due to the additional complexity of managing both stroke and chronic conditions. Another prior study has suggested that higher expenditures on prescription drugs are the costliest contributor to total excess expenditures associated with chronic conditions [14].

Second, the OOP healthcare expenditures of stroke survivors comorbid with chronic conditions showed a trend of rising volatility. This phenomenon can be explained by the evidence that, with the increasing need for high quality of care, surgical clipping and intravascular intervention which can prevent stroke recurrence are becoming more popular, but the costs are highly priced and far exceeding the ceilings of the reimbursement [22]. Moreover, since the National Health Commission limits the proportion of consumable costs, some imported consumables are excluded from the catalogue of social health insurance reimbursement and are fully paid by patients. Additionally, there have been a rising prevalence of hemorrhagic strokes rather than ischemic strokes, and majority of hospitalization costs for hemorrhagic stroke may be the OOP payment, while this proportional is lower for ischemic strokes [23]. Thus, the fluctuant rising OOP healthcare expenditures could be related to the treatment of stroke with different subtypes. The likelihood of patients with hemorrhagic stroke treated in the intensive care unit (ICU) was higher than that for ischemic stroke patients. The copayments of ICU are commonly higher than that of ordinary wards, since many commonly prescribed neurologic medications used in ICU are self-paid [22].

Third, incremental healthcare expenditures attributable to chronic conditions can be driven by more utilization of inpatient and outpatient treatments. Such higher utilization could be due to the development of health insurance. Our findings show that the rate of catastrophic payments decreased during the past decade, suggesting that health insurance protects families against financial hardship. Nevertheless, the total healthcare expenditures increased, posing a considerate burden on health systems. The rapid development of China’s social health insurance system in terms of population coverage and benefits, has contributed to boosting health care demand, especially after the 2009 health system reform [24]. The annual hospitalization rate tripled from 4% in 2000 to 13% in 2015 [25]. One previous study has showed that increases in reimbursement rate of social health insurance at county hospitals is associated with about 20% greater probability of visiting such hospitals in China [26]. Stroke survivors with diabetes had about 20–40% more inpatient stays and outpatient visits than those without diabetes [14].

Moreover, we found that economic burdens were higher for those patients with multiple chronic conditions than those persons with any single condition. The finding is consistent with the prior study, showing that there have been biological and psychosocial mechanisms explicating how stoke leads to diabetes mellitus and impaired cognitive function, and concomitant chronic conditions are interlinked [27]. Economic burden increase with the number and severity of concomitant chronic conditions. Our findings highlighted the complexities and need for care coordination for people with multimorbidity, given evidence that patients who receive care for a single chronic condition may not receive care for other unrelated conditions [28].

The increased prevalence and burden of comorbid chronic conditions among stroke survivors highlight the importance of healthcare management in improving quality of life and averting the economic burden. Integrated people-centered healthcare is an approach to improving quality of life and health outcomes, while reducing healthcare use and expenditures. As part of the national strategy named Healthy China 2030, the national government released a strategic framework on integrated healthcare that are delivered in a way that ensures people receive a continuum of prevention, diagnosis, treatment, disease management and rehabilitation according to their needs, at different levels and sites of care within the health system. Several pilots are being implemented at the local level. For example, Chinese Stroke Center Alliance developed a nationwide, hospital-based, regional collaborative stroke networks to improve quality of care through integrating community-, prehospital- and hospital-care [29]. In addition, community-based chronic condition self-management programs liked to clinical services can give stroke survivors with multimorbidity the knowledge and skills to manage their conditions. Effective self-management programs can improve quality of life and health outcomes, while reducing healthcare use [30]. Moreover, payments to incentivize coordination should be applied over care episodes, reduce spending, and improve quality [31]. Prior study has highlighted post-acute spending as a potential target for bundled payment schemes [32]. The results show that the fixed diagnosis-related group payment has likely contributed to reducing the length of stay and containing spending within 30 days after admission.

The strengths of our study include evaluation of a 10-year longitudinal study and a nationally representative sample of stroke survivors’ chronic comorbidities. Several limitations merit comment. First, the retrospective trend analysis limited the ability to draw any causal inference from the findings. Second, self-reported healthcare expenditure may lead to recall bias. Third, the use of self-reported measures of comorbidities may underestimate their prevalence, particularly for older persons and those from lower socio-economic and educational backgrounds who may be more likely to under-report. Fourth, we examined the effects of multimorbidity on healthcare expenditures, by simply counting the number of four chronic diseases without accounting for the different clusters and severity of chronic diseases. Fifth, due to the unavailability of data, the information on mortality can not be access, so this study can not be able to analyze the opportunity cost regarding stroke comorbid with chronic conditions. Sixth, there may be some other contributory factors not considered in this analysis that influenced the healthcare expenditures, including stroke type and treatment therapies. It is warranted to identify more comorbidities among stroke survivors and apply unequal weights according to the type and severity of chronic conditions to explore the effect of multimorbidity. Further research is also needed to understand the economic burden by healthcare components (e.g. inpatient stays, emergency room visits, outpatient visits, and prescription drugs), and incidence-based studies coupled with modelling techniques are also needed to estimate lifetime costs of stroke. Such studies will give a better understanding for evaluating whether improvements in the approach to treatment and management of multiple chronic conditions will translate to reduced expenditures in this population.

## Conclusion

Concurrent chronic conditions are an important driver of healthcare expenditures in patients following a diagnosis of stroke. Increased efforts in integrated healthcare with a continuum of prevention, diagnosis, treatment, disease management and rehabilitation, can be effective in managing multimorbidity, reducing medical expenditures, among survivors of stroke.

## Data Availability

This research uses data from China Health and Nutrition Survey (CHNS), which is available online (https://www.cpc.unc.edu/projects/china).
